# Understanding health care provider barriers to hospital affiliated medical fitness center facility referral: a questionnaire survey and semi structured interviews

**DOI:** 10.1186/s12913-017-2474-y

**Published:** 2017-08-03

**Authors:** Carissa Smock, Sonia Alemagno

**Affiliations:** 1 Case Western Reserve University, 11100 Euclid Avenue MS 6036, Cleveland, OH 44016 USA; 2 Kent State University College of Public Health, 326 Lowry Hall, 750 Hilltop Dr, Kent, OH 44709 USA

**Keywords:** Medical fitness, Exercise referral, Health care provider, Integrated facility, Medical fitness center facility

## Abstract

**Background:**

The purpose of this study is to understand health care provider barriers to referring patients to Medical Fitness Center Facilities within an affiliated teaching hospital system using referral of diabetic services as an example. The aims of this study include: (1) to assess health care providers’ awareness and use of facilities, (2) to determine barriers to referring patients to facilities, (3) identify current and needed resources and/or changes to increase referral to facilities.

**Methods:**

A 20-item electronic survey and requests for semi-structured interviews were administered to hospital system directors and managers (*n* = 51). Directors and managers instructed physicians and staff to complete the survey and interviews as applicable. Perceived barriers, knowledge, utilization, and referral of patients to Medical Fitness Center Facilities were collected and examined. Descriptive statistics were generated regarding practice characteristics, provider characteristics, and referral.

**Results:**

Of the health care providers surveyed and interviewed (*n* = 25) 40% indicated verbally suggesting use of facilities, 24% provided a flyer about the facilities. No respondents indicated that they directly referred patients to the facilities. However, 16% referred patients to other locations for physical activity - including their own department’s management and prevention services. 20% do not refer to Medical Fitness Center Facilities or any other lifestyle programs/locations. Lack of time (92%) and lack of standard guidelines and operating procedures (88%) are barriers to referral. All respondents indicated a strong ability to refer patients to Medical Fitness Center Facilities if given education about referral programs available as well as standard clinical guidelines and protocol for delivery.

**Conclusion:**

The results of this study indicate that, although few healthcare providers are currently referring patients to Medical Fitness Center Facilities, health care providers with an affiliated Medical Fitness Center Facility not only want clinical standard guidelines, protocol, and training to refer patients to Medical Fitness Center Facilities, but believe they have the ability to increase referral if given these tools. The Medical Fitness Association has a unique opportunity to bridge health care providers to Medical Fitness Center Facilities by developing clinical practice guidelines in cooperation with the American Diabetes Association.

**Electronic supplementary material:**

The online version of this article (doi:10.1186/s12913-017-2474-y) contains supplementary material, which is available to authorized users.

## Background

### What are medical fitness center facilities

The Medical Fitness Association (MFA) is a non-profit, professional membership organization whose mission is to “ascertain and respond to the needs of medically integrated centers throughout the world” [1]. MFA publishes medical fitness standards and guidelines for medical fitness center facilities (MFCF) available for purchase and offers a Medical Fitness certification to facilities that adhere to these standards and guidelines. According to the MFA, the factors that define certified MFCF include: 1) consistent and ongoing medical supervision, 2) staff who are certified and licensed, 3) member safety policies and actionable procedures, 4) programs including disease prevention and management, 5) programs including therapeutic-lifestyle and health-risk reduction, 6) member-specific health screening, 7) fitness measurements and logs, 8) measurable outcomes including quality, and 9) attention to improving the community’s health [1].

Research has yet to be completed to determine the true value of MFCF in terms of increasing patient or member physical activity. However, the Patient Protection and Affordable Care Act movement to accountable care and population health management is prompting US hospitals to extend services beyond the sick care model and buy or construct fitness centers. Hospitals rebrand the fitness centers to MFCF and affiliate them with their hospital. The MFA reports that the number of MFCF has grown from 79 in 1985 to 1284 in 2014 with membership to facilities of more than 4 million people. Financially, MFCF’s were designed to protect a hospital’s market share as members pay out-of-pocket rather than through insurance for retail-oriented wellness services and sponsorships [2].

MFCF’s attract a unique population that are at increased risk of morbidity complications and mortality without an increase in physical activity [3]. The average age of members is older than the usual health club member at about 50 years of age, and half have never been a member of an exercise facility before their membership to a MFCF [2]. Half of the members are managing at least one chronic condition. Of these members, 30–35% report using a physician or other clinical services of the affiliated hospital for the first time [2].

### What we know about health care providers who refer to MFCF’s

Despite the availability of programs [4], providers cite that they do not have the training necessary to use these programs and make exercise referrals. Many providers are not aware of these programs at all [5, 6]. Currently provider incentives have yet to align with exercise referrals. Provider reimbursements, credentialing, licensure, periodic schedules, value-based purchasing, meaningful use, and pay-for performance must shift to support exercise referrals [7].

Previous evaluations of provider attitudes toward provider involvement in exercise treatment have focused on the use of patient counseling and varied definitions of exercise referrals and prescriptions [8–11]. Participation in training programs specifically designed to help providers construct exercise referrals is generally correlated with greater acceptance of provider-initiated exercise referral interventions [12]. Overall, research suggests that professional support of provider initiated exercise interventions increases organizational policy and professional norms that support active involvement in exercise prescription policies and procedures [8].

### The US compared to other countries

Over the past couple of decades, Exercise Referral Schemes (ERS) have become one of the most common interventions across the UK used to promote physical activity in primary care with now over 600 ERS; however, documentation of ERS dates back almost 90 years. Inactive adults within primary care settings are identified and referred to third party services. The service then prescribes a patient-specific exercise program and tracks results [13, 14].

Given the provisions of the PPACA in the US, hospitals are encouraged to develop and implement policies and programs that prevent chronic diseases [15]. The MFA developed chronic disease prevention standards to guide programing. Hospital systems that own MFCF are therefore in a unique position to include ERS internally to its patients. Despite this opportunity, health care providers do not have the training to facilitate ERS - largely because the American Board of Medical Specialties (ABMS), the principle certification body, opted out of accrediting a lifestyle-based subspecialty. ABMS cites opting out of lifestyle-based education because of the low number of candidates who registered for the exam, in addition to unstandardized lifestyle programs [6]. Although there is not an ABMS lifestyle specialty, there are 13 non-accredited fellowship training programs and board certifications from organizations such as the American Board of Provider Nutrition Specialists and the American Board of Obesity Medicine. Without an accredited subspecialty, medical professionals are less likely to seek advanced training in these areas, medical schools have less incentive to teach this material, and students have less incentive to demand greater instruction. Because insurance reimbursements are geared toward treatment, there are few financial incentives for healthcare providers to offer exercise and lifestyle interventions. Additionally, providers may not know what physical activity interventions to prescribe, or where to send their patients [6].

Due to these challenges, MFA is part of a collaboration with American College of Sports Medicine (ACSM), and the American Council on Exercise (ACE) to focus on better integration of physical activity into the nation’s healthcare through the Exercise is Medicine (EIM) initiative. The goal of this collaboration is to advocate for physical activity to be included in health providers’ standards of care [16]. In order to include physical activity as a standard of care patient physical activity needs to be assessed and recorded as a vital sign. Patients must then be referred to certified MFCF and programs based on their needs. In the United States 20 health systems are in various stages of transitioning to use of exercise as medicine. These systems are: 1) Including EIM as required curriculum in all four years of medical school, 2) Using electronic medical records (EMR) to collect measures of physical activity as a vital sign, 4) Using patient education material with EMR’s, and 4) hiring an EIM Coordinator [16].

## Methods

The purpose of this exploratory study is to describe provider barriers to referring patients to Medical Fitness Center Facilities (MFCF) within an affiliated teaching hospital system. The hospital systems’ referral of diabetic services was examined as an example. Increased diabetes prevention and management was identified as a wellness initiative in this hospital’s Community Health Needs Assessment (CHNA) and strategic plan. Referral to MFCF seems to be limited across the hospital system and there is limited information about attitudes towards referring patients to these facilities. The aims of this study include: (1) to assess health care providers’ awareness of and use of MFCF’s, and (2) to determine the barriers to referring patients to MFCF. The results of this study are anticipated to inform researchers of current and needed resources and/or changes within the hospital system that might be considered to increase referral of patients to MFCF’s.

### Study location and participants

The research was conducted in a 500 plus-registered-bed, teaching hospital staffed by more than 1000 physicians, and over 3000 healthcare professionals and support staff who serve a population of more than 1.2 million people. The hospital system has multiple membership-based fitness facilities. Potential MFCF referral opportunities across the hospital system were identified by interviewing hospital system directors and managers. These directors and managers identified those who met the inclusion criteria of providing primary care to diabetic patients and have the potential to refer patients to the MCFC. Exclusion criteria included staff and physicians of the hospital system who do not have contact with diabetic patients and therefore do not have the potential to refer patients to the MCFC.

### Survey development

Survey content and methodology were developed and refined using input and feedback from both clinical and academic partners. The survey entitled *Provider medical fitness center facility referral survey* is included for reference Additional file 1 and consists of short answer, multiple choice, and Likert-scale questions. Survey constructs include current MFCF referral practices, awareness of opportunities to refer patients to MFCF, attitudes toward referring patients to MFCF, perceived barriers to MFCF referral, and a needs assessment for standard guidelines and training for MFCF referral.

Draft survey instruments were distributed to these partners, and feedback was gathered through email comments as well as through in-person discussion. The clinical and academic partners institutional review boards approved the final version of the survey instrument and methodology.

### Data collection

A 20-item electronic survey was administered to the hospital system directors and managers of diabetes services (*n* = 51). Directors and mangers then instructed physicians and staff to complete the survey as applicable. Semi-structured interviews were also conducted using the *Provider medical fitness center facility referral semi-structured interview guide* included for reference Additional file 2. Twenty-five of 51 surveys were returned over the course of data collection (a response rate of 49.02%); two emails were incorrect and did not go through and semi-structured interviews were conducted resulting in a final *n* = 25. Directors, managers, physicians, and staff (*n* = 25) perceived barriers, knowledge, utilization, and referral of patients to MFCF were collected and examined. The survey was generated in Qualtrics and designed to capture information regarding patient referral to MFCF. These directors and managers forwarded the email to their staff member best qualified to answer the questions and often scheduled in-person, semi-structured interviews to answer the questions. The planning department reminded directors to participate in the survey throughout the following week after the initial survey distribution to increase the response rate.

### Data analysis

Once surveys were completed in Qualtrics, descriptive statistics were generated for questions regarding practice characteristics, provider characteristics, and MFCF referral. Since the physicians and staff are all operating under the same health system, the quality of chronic care management should be similar. Also, the dialogues were used to inform the researcher about currently available and needed clinical standard guidelines and training for improving referral to MFCF.

## Results

### Characteristics of participants

Of the participants, 36% were male, 64% were female, 92% were white, and 8% percent were African American. The participants’ highest level of education included 40% with a bachelor’s degree, 32% with a master’s degree, and 28% with a doctoral degree.

Respondents represented a relatively even distribution between the various diabetes prevention and management services and centers offered by the hospital system (based on how many employees work at each location) with main campus including 48%, fitness facility 32%, a rural hospital 8% and 12% coming from various locations through the hospital. Represented medical and administrative specialties and program management included inpatient, health and wellness, rehabilitation, visiting nurse services/home health, rural diabetes/prevention programs, diabetes center, clinical trials/research, endocrinology, primary care, and senior services. About half of the of the physicians and staff reported practicing for 14 years or longer, with 25% stating that they had been practicing for at least 20 years (see Table 1).

**Table 1:** Characteristics of Participants (*n* = 25)

	Number	Percent
Gender		
Male	9	36
Female	16	64
Service Administration Location		
Main Campus	12	48
Medical Fitness Center Facility	8	32
Rural hospital	2	8
Other	3	12
Occupation		
Nursing	8	32
Doctor/Research	7	28
Business/Professional	10	40
Education		
Bachelor’s Degree	10	40
Master’s Degree	8	32
Doctoral Degree	7	28
Race/Ethnicity		
White Non-Hispanic	23	92
Black	2	8
Years of service		
Less than 14	13	52
> 14–20	6	24
> 20	6	24

Forty percent of participants indicated that they, or those in contact with patients in their department, verbally suggested use of the MFCF, 24% provided a flyer about the MFCF. No respondents indicated that they directly referred patients to the MFCF. Several departments had their own management and prevention services that they utilized instead with 16% indicating that they referred patients to these other services rather than the MFCF. Twenty percent do not refer to MFCF or any other lifestyle programs/locations. Forty-eight percent indicated that they provided education about physical activity, 36% included education about healthy diet, and 24% including stress management.

### Barriers to referring

Despite lack of perceived benefits of MFCF, concerns regarding referral were reported. Lack of time (92%), standard clinical guidelines (88%), patient compliance (32%), lack of awareness/need for education about programs (40%), were commonly cited as barriers referring patients to MFCF. Methods of reporting use of MFCF programs varied widely from no reporting methods to various electronic systems and metrics.

### Needs assessment

Finally, the survey asked participants open-ended questions including if there were any successes, concerns or opportunities that they believe would impact their practice. All respondents indicated a strong ability to refer patients to MFCF if given education about available programs as well as protocols for referral. Need for internal and external communication resources to decrease duplication of services and silos was indicated as well as financial and capacity barriers to access of the fitness facilities (see Table 2).

**Table 2:** Lifestyle Service Administration (*n* = 25)

	Number	Percent
Physical Activity	9	36
Diet	12	48
Stress	6	24
How and why diabetes develops	4	16
MFCF referral currently administered		
Provider verbally mentions MFCF	10	40
Providers gives patients an MFCF handout	6	24
Provider sets up appointments for patients at MFCF	0	0
Provider refers patients to other lifestyle programs (not MFCF)	4	16
Provider does not refer to MFCF or any other lifestyle programs/locations	5	20
Ability to refer patient to MFCF		
Very Strong	21	82
Strong	4	16
Decent	0	0
Working on it	0	0
Challenging	0	0
Barriers to referral to MFCF		
Lack of provider time	23	92
Lack of standard guidelines/ system in place	22	88
Patient compliance	8	32
Lack of awareness/education	10	40
Providers think patients do not have the means to go to MFCF	4	16
Requested assistance with MFCF referral guidelines education	25	100
Requested assistance with implementing MFCF referral	25	100

### Limitations

This study had several limitations, with the first being its small sample size (*n* = 25). However, the response rate is excellent for physician and medical staff surveys [17]. The sample enabled description of staff and physicians’ attitudes, perceptions, and familiarity with referral to MFCF therefore adding to the scarce literature in this area. The importance of physicians’ and staff perceptions of MFCF is crucial as they provide the majority of diabetes care. Secondly, nonresponse bias is always a research concern (the response rate was 49.02%). However, response rate is in line with what has been reported by national surveys of physicians and clinical staff that ranged from 25.6% to 70% [17].

## Discussion

Physicians and other professional staff working with diabetic patients are not frequently referring to MFCF. However, with the development of standard clinical guidelines and hospital specific standard operating procedures there is potential for regular referral to MFCF. These guidelines and procedures have potential to integrate patients into the growing numbers of MFCF. Stronger links between the healthcare system and MFCF can help the medical community promote prevention activities that need to become a permanent part of the patient’s life.

While there is evidence that patient counseling is generally accepted within the provider community, the variety of provider -initiated exercise referral interventions are highly varied [8, 8, 9, 11]. Given a growing body of research that suggests the potential economic and health benefits of detailed exercise referral, it is important to evaluate provider acceptance of these provider -initiated exercise referral interventions [8–11].

Due to the leadership role of the MFA in the MFCF industry and collaborations with the EIM initiative, the MFA has a unique opportunity to bridge health care providers to MFCF by developing the needed standard clinical guidelines (SCG) in cooperation with the American Diabetes Association (ADA) rather than recommending each MFCF create these SCG independently. Should the MFA include the SCG for referral as part of their certification process, approved centers would then connect the health care providers to the treatment solution of diabetes. SCG also need to be developed in cooperation with the American Heart Association (AHA) for heart disease and stroke.

In order to create SCG, the MFA should examine the limited yet available data from all of its certified MFCF referral programs to determine which programs align with ADA and AHA guideline lines as well as to test which programs result in sustained patient PA behaviors. Although the sample sizes may not allow for statistical significance, they still provide a pilot test, starting point, and an opportunity to increase sample size, validity, and reliability.

## Conclusions

The results of this exploratory study indicate that, although few healthcare providers are currently referring patients to MFCF, health care providers with an affiliated MFCF not only seem to want CSG that include ERS to MFCF, but believe they have the ability to increase referral to MFCF if given these SCG along with training, time, and promotional tools. Therefore, this study suggests SCG’s that include ERS may need to be created and offered because these guidelines have potential to increase heath care provider referral of patients to MFCF and ultimately increase use of MFCF by patients. The MFA has a unique opportunity to bridge health care providers to MFCF by developing these SCG in cooperation with the ADA. Next steps for research include 1) conducting this study across multiple sites to increase sample size 2) creating standard clinical guidelines, operating procedures, training, and promotional tools of ERS to MFCF and 2) pre-and post training evaluation of ERS to MFCF.

## Additional files

Additional file 1: Survey_Provider medical fitness center facility referral. Provider medical fitness center facility referral survey. Survey distributed to health care providers collect data about their referral to medical fitness center facilities. (DOCX 16 kb)

Additional file 2: Interview guide_Provider medical fitness center facility referral. Provider medical fitness center facility referral semi-structured interview guide. Guide used to conduct semi-structured interviews with health care providers collect data about their referral to medical fitness center facilities. (DOCX 16 kb)

## Abbreviations

ABMS: American Board of Medical Specialties
ACSM: American College of Sports Medicine
ADA: American Diabetes Association
AHA: American Heart Association
CCT: Compulsory Competitive Tendering
EIM: Exercise is Medicine
EMR: Electronic Medical Record
ERS: Exercise Referral Schemes
EVS: Exercise as a Vital Sign
MFA: Medical Fitness Association
MFCF: Medical Fitness Center Facilities
NDPP: National Diabetes Prevention Program
PA: Physical Activity
PREP: Physical Referred Exercise Program
SCG: Standard clinical guidelines
