# Supplementary material for: Understanding health care provider barriers to hospital affiliated medical fitness center facility referral: a questionnaire survey and semi structured interviews
Source: BMC Health Serv Res. 2017 Aug 3;17:520. doi: 10.1186/s12913-017-2474-y (PMC5543749; doi:10.1186/s12913-017-2474-y)
Supplement: Supplementary file 1 — Survey_Provider medical fitness center facility referral. Provider medical fitness center facility referral survey. Survey distributed to health care providers collect data about their referral to medical fitness center facilities. (DOCX 16 kb) [file 12913_2017_2474_MOESM1_ESM.docx]

Provider medical fitness center facility referral survey

Q1 Did PhD candidate, Carissa Smock, meet with or shadow you specifically?

- Yes
- No
- Don’t know

Q2 What is your gender?

- Male
- Female
- Prefer not to answer

Q3 What is your racial/ethnic background? Please select all that apply:

- White
- Black or African American
- American Indian or Alaska Native
- Asian/Pacific Islander
- Hispanic or Latino
- Prefer not to answer

Q4 What is your highest level of education?

- High School
- Some college credit, no degree
- Trade/technical/vocational training
- Associate degree
- Bachelor’s Degree
- Master’s Degree
- Decorate Degree

Q5 Where do you personally deliver service? Please select all that apply:

- Main Campus
- Medical fitness center facility
- Rehabilitation hospital
- Visiting nurse services
- Rural hospital
- Anywhere else? ____________________

Q6 What types of diabetes service do you deliver? Please select all that apply:

- Diet
- Physical activity
- Stress management
- How & why diabetes develops
- Any other services? ____________________

Q7 What is your role in diabetes services? Please select all that apply:

- Primary Care Physician
- Pharmacist
- Diabetes Nurse
- Diabetes Nurse Practitioner
- Endocrinologist
- Dietitian
- Diabetes Educator
- Administration/management
- Any other role? ____________________

Q8 How many YEARS (please round to the nearest year) have you delivered your current service(s)? ­­­­­­­­______________

Q9 Does your practice refer patients to the Medical Fitness Center Facilities (MFCF)?

- I/we verbally mention the MFCF
- I/we give patients an MFCF handout
- I/we set up appointments for patients at MFCF
- I/we refer patients to other lifestyle programs (not MFCF)
- I/we do not refer to MFCF or any other lifestyle programs/locations
- Any other way? ____________________

Q10 Please rate your ability to refer patients to the Medical Fitness Center Facilities (MFCF).

- Very Strong
- Strong
- Decent
- Working on it
- Challenging

Q11 What roadblocks are getting in the way of referring patients to the Medical Fitness Center Facilities (MFCF)?

- Lack of my time
- Lack of standard guidelines/ system in place
- Lack of patient compliance
- Lack of awareness/education
- I think patients do not have the means to go to MFCF
- Something else? ____________________

Q12 What might increase your ability to refer patients to the Medical Fitness Center Facilities (MFCF)?

- Assistance with MFCF referral guidelines/education
- Assistance with implementing MFCF referral
- Something else? ____________________

Q13 Is there anything you'd like to share - successes, concerns or opportunities that you believe will impact your practice? ____________________________________________________________________________________________________________________________________________________________________________________________________________________________________

Thank you for helping to ensure our hospital system delivers the most effective system of diabetes and prevention services possible - and a successful experience for our PhD's!  Your feedback will be confidentially reviewed to improve quality and learning! Please email examples of materials you use to deliver your services to carissa.smock@XXX.org.
