# Supplementary material for: Understanding health care provider barriers to hospital affiliated medical fitness center facility referral: a questionnaire survey and semi structured interviews
Source: BMC Health Serv Res. 2017 Aug 3;17:520. doi: 10.1186/s12913-017-2474-y (PMC5543749; doi:10.1186/s12913-017-2474-y)
Supplement: Supplementary file 2 — Interview guide_Provider medical fitness center facility referral. Provider medical fitness center facility referral semi-structured interview guide. Guide used to conduct semi-structured interviews with health care providers collect data about their referral to medical fitness center facilities. (DOCX 16 kb) [file 12913_2017_2474_MOESM2_ESM.docx]

**Provider medical fitness center facility referral semi-structured interview guide**

Q1 Where do you personally deliver service at the hospital

Probe: This could be main campus, a medical fitness facility, the rehabilitation hospital.

Q2 What types of diabetes service do you deliver?

Probe: These could be prevention or disease management services

Prompts: Can you give some examples of how you deliver these services?

Are these services held at specific locations?

Are these services held by specific people?

Q3 What is your role in delivering the services you described?

Probe: Program manager, coordinator, educator.

Prompt: Are there other roles you take on?

What causes you to take on these other roles?

Q4 Does your practice refer patients to the Medical Fitness Center Facilities (MFCF)?

Probe: Verbally, via handouts, appointment set-up

Prompt: Are there other services you refer patients to for diabetes prevention/ management?

Q5 How would you rate your ability to refer patients to the Medical Fitness Center Facilities (MFCF).

Probe: Very strong, strong, decent, you’re working on it, or is it challenging?

Prompt: What do you think could change this rating?

Q6 What do you believe some of the roadblocks are in referring patients to the Medical Fitness Center Facilitates?

Probe: Lack of time, guidelines, systems, patient compliance, awareness, education, funding.

Prompt: Do you think these barriers can be overcome?

Q7 What do you think might increase your ability to refer patients to the Medical Fitness Center Facilities (MFCF)?

Probe: Assistance with MFCF referral guidelines/education, assistance with implementing MFCF referral

Prompt: Do you think it is feasible to implement a referral system with these tools?

Q8 Is there anything you'd like to share - successes, concerns or opportunities that you believe will impact your practice?
